# Kinetics and magnitude of viral RNA shedding as indicators for Influenza A virus transmissibility in ferrets

**DOI:** 10.1038/s42003-023-04459-0

**Published:** 2023-01-23

**Authors:** Joanna A. Pulit-Penaloza, Nicole Brock, Jessica A. Belser, Xiangjie Sun, Claudia Pappas, Terrence M. Tumpey, Taronna R. Maines

**Affiliations:** Influenza Division, National Center for Immunization and Respiratory Diseases, Centers for Disease Control and Prevention, Atlanta, GA USA

**Keywords:** Influenza virus, Experimental models of disease

## Abstract

The ferret transmission model is routinely used to evaluate the pandemic potential of newly emerging influenza A viruses. However, concurrent measurement of viral load in the air is typically not a component of such studies. To address this knowledge gap, we measured the levels of virus in ferret nasal washes as well as viral RNA emitted into the air for 14 diverse influenza viruses, encompassing human-, swine-, and avian-origin strains. Here we show that transmissible viruses display robust replication and fast release into the air. In contrast, poorly- and non-transmissible viruses show significantly reduced or delayed replication along with lower detection of airborne viral RNA at early time points post inoculation. These findings indicate that efficient ferret-to-ferret transmission via the air is directly associated with fast emission of virus-laden particles; as such, quantification of viral RNA in the air represents a useful addition to established assessments of new influenza virus strains.

## Abstract (web summary)

The analysis of viral RNA shedding and emission dynamics for 14 diverse influenza viruses in ferrets reveals that efficient ferret-to-ferret transmission via air is directly associated with robust emission of virus-laden particles at early times.

## Introduction

There are three prerequisites for an influenza virus to cause a pandemic. The virus must be antigenically distinct from human influenza viruses to which we have immunity, be able to infect and efficiently replicate in a human host, and possess molecular features facilitating sustained transmission among people. Zoonotic influenza viruses occasionally cross species barriers to cause human infection; however, despite a lack of preexisting immunity in the human population to most of these viruses, subsequent human-to-human transmission is typically not observed^1^. The ferret model is currently the most valuable tool to study the transmissibility of influenza viruses in a laboratory setting, in that they are naturally susceptible to influenza virus infection, can display pronounced clinical signs of infection, and can transmit virus to naïve animals^2^. The Respiratory Droplet Transmission (RDT) model involves housing of infected and naïve ferrets in adjacent cages, which allows for the exchange of air through perforated cage walls but eliminates direct and indirect contact between animals, facilitating airborne-only transmission (inclusive of droplet and droplet nuclei transmission). The Direct Contact Transmission (DCT) model involves housing an infected ferret in the same cage as a naïve ferret, facilitating any mode of transmission (direct, indirect, and/or via the air)^3^. Human adapted H1 and H3 subtype viruses typically transmit efficiently between ferrets in both models^4,5^. In contrast, zoonotic influenza viruses, including those isolated from humans, display a variety of transmission profiles in the ferret model. Generally, H5 subtype viruses are unable to transmit, whereas H7 and H9 viruses vary from those that are unable to transmit in direct contact setting to those that are capable of transmission in either model^6^. Swine-origin H1 and H3 viruses typically transmit efficiently between cohoused ferrets but less frequently via air, but in rare cases, transmission occurs as efficiently as human seasonal viruses^7–9^.

Scientists undertake tremendous efforts to routinely monitor and characterize newly emerging viruses for pandemic preparedness, inclusive of research conducted to identify strain specific differences in transmission profiles and the mutations required for adaptation to a new host^10^. There is also significant interest in elucidating the kinetics of virus shedding following infection and quantifying viral load released into the air; an area of research that is understudied due to practical and methodological challenges. Although most bioaerosol sampling devices are suitable for collection and study of pathogens aerosolized in laboratory settings, efficient collection of virus-laden particles emitted (at much lower rate and concentration) from experimentally inoculated animals is challenging^11–13^. Range of instruments including aerosol particle counters, cyclone samplers, and cascade impactors, have been used to evaluate size-distribution and the quantity of influenza virus emitted into the air by inoculated ferrets^14–17^. These studies support that particle size represents an important consideration in virus transmission, with most of the viral RNA and infectious virus found in particles larger than 1.5 µm and transmission via the air largely mediated by particles in that size range^14,15^. While the size of virus-laden particles affects the initial site of virus deposition, transmission can only occur if the virus can bind to the epithelial cells at the deposition site^18,19^. Upper respiratory tract epithelium that is enriched in alpha 2,6-linked sialic acid (α2,6 SA) receptors has been recognized as a critical determinant for influenza virus transmission^20,21^. In agreement, human-adapted influenza viruses which preferentially bind α2,6 SA receptors are capable of efficient replication at that site, in contrast to avian-origin viruses which preferentially bind alpha 2,3-linked sialic acid (α2,3 SA) receptors^22,23^. Previous studies reported a link between viral gene constellations, the quantity of virus-laden particles released from infected ferrets, and airborne transmission^15,16^. Consistent with this observation, avian H5N1 influenza viruses, which were not capable of airborne transmission in ferrets, were detected in breath and sneeze collected from anaesthetized ferrets at lower quantities as compared to human-adapted viruses that transmitted well in this model^14^ highlighting the need for more extensive comparative analysis encompassing wider variety of IAV strains.

In this study, we sought to expand on previous analyses by employing a wide range of human-adapted, swine-origin, and avian-origin influenza A viruses (IAVs), with diverse gene constellations and mammalian transmissibility phenotypes. Airborne particles were collected from unanesthetized ferrets using a National Institute for Occupational Health (NIOSH) bioaerosol sampler that fractions air particles into three sizes (>4, 1–4, <1 µm)^24^, followed by real-time RT-PCR quantification of influenza virus M segment RNA. To better understand the link between virus release into the air and airborne transmission, the majority of these experiments were performed concurrent with pathogenesis and transmission assessments. We found that for viruses capable of transmitting through the air between ferrets, significantly higher levels of viral RNA were detected in both nasal wash and air samples, and detection was typically at earlier days post-inoculation compared with viruses that were not capable of efficient airborne transmission. These data indicate that robust replication in the upper respiratory tract of ferrets and early release of virus into the air represent important factors required for efficient transmission.

## Results

### Comparison of viral RNA shedding following inoculation of ferrets with IAVs displaying diverse transmissibility profiles

**Fig. 1:**
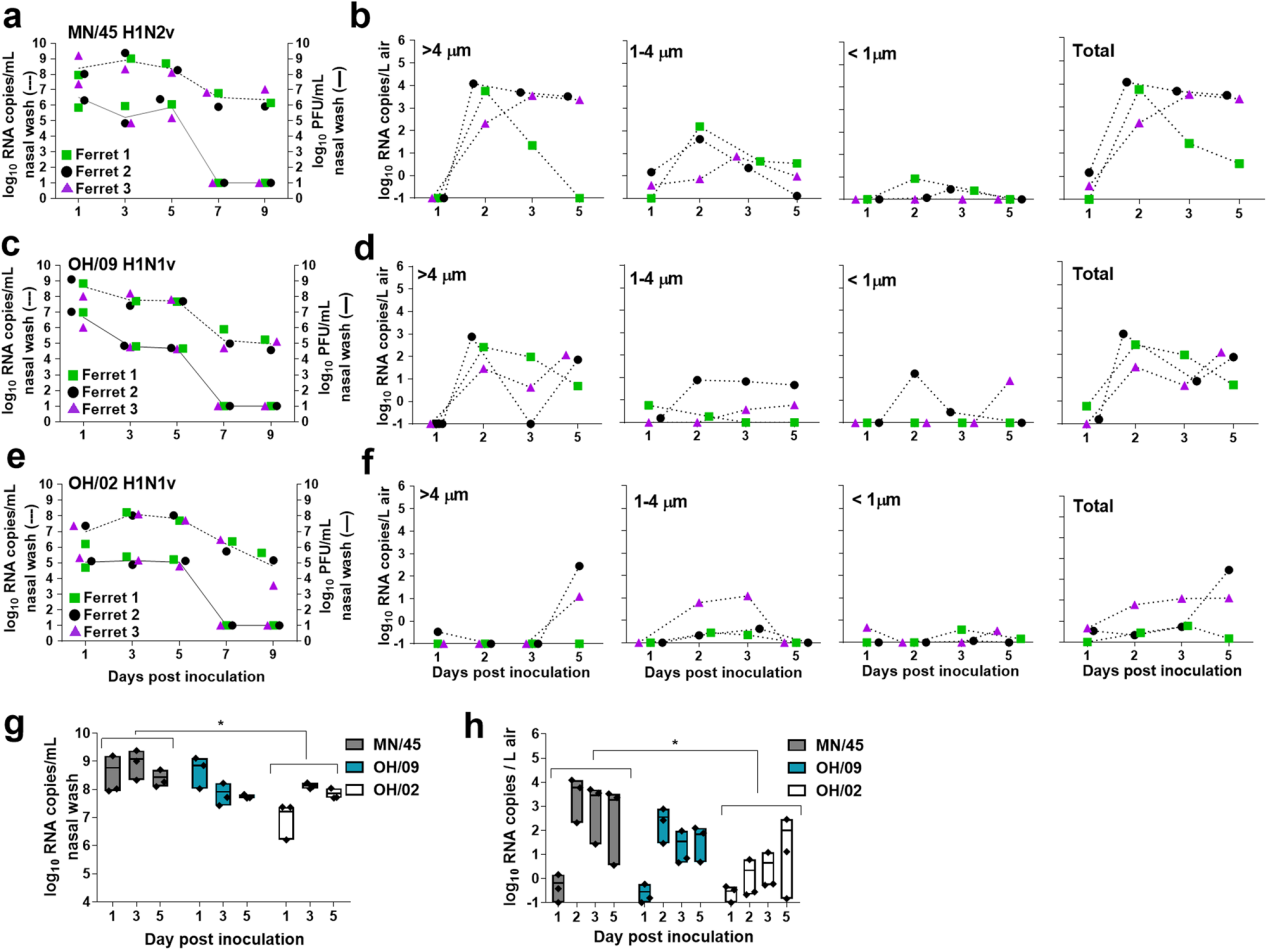
Influenza A viral RNA measured in the air following inoculation of ferrets. Nasal wash specimens from ferrets (*n* = 3 per virus) inoculated with A/Minnesota/45/2016 A(H1N2)v **a**, A/Ohio/09/2015 A(H1N1)v **c**, and A/Ohio/02/2007 A(H1N1)v **e** were collected on days 1, 3, 5, 7, 9 p.i. Infectious virus was quantified by standard plaque assay (solid lines) and viral RNA copies were quantified using real time qRT-PCR (dashed lines). Individual ferrets are shown as different symbols (ferret 1-green square, ferret 2-black circle, ferret 3-purple triangle). Limit of detection was 1 log_10_ PFU/ml or RNA copies/ml of nasal wash. Air samples were collected from each ferret cage for 2 hours on indicated days using a two-stage cyclone sampler which fractioned particles by size: >4 µm, 1–4 µm, <1 µm. Viral RNA in each sample was quantified using real time qRT-PCR and the data for each virus; A/Minnesota/45/2016 A(H1N2)v **b**, A/Ohio/09/2015 A(H1N1)v **d**, A/Ohio/02/2007 A(H1N1)v **f**, and individual ferret is shown. Limit of detection was 21 copies per 210 liters of air collected at each time point (0.1 RNA copy/L of air). Summary figures comparing viral RNA copies detected in the nasal wash specimens **g** and in the air **h** collected through day 5 p.i. The points on graphs are the data for individual ferrets, the bars represent minimum and maximum, and the lines represents the mean. Area under the curve for each of these time courses was calculated and statistical significance was evaluated using One-Way ANOVA (Tukey’s post-test [g]) and Kruskal-Wallis test (Dunn’s post-test [h]). *p < 0.05.

The 2009 A(H1N1)pdm09 influenza pandemic highlighted the potential of swine-origin influenza viruses to cause human disease and global spread. Since then, there has been an increased focus on IAV surveillance and mammalian transmissibility assessments using the ferret model. Swine-origin influenza viruses typically transmit easily between cohoused ferrets but differ widely in their ability to transmit between ferrets in setting where direct and indirect contact (fomites on intermediate surfaces) are eliminated, and transmission can only occur via air^7,9^. Unfortunately, routine ferret transmission studies do not include assessments of the kinetics and levels of virus released into the air by infected animals. To address this gap in knowledge we analyzed virus levels in nasal washes concurrent with quantification of viral RNA levels in the air. We selected three H1 subtype swine-origin influenza viruses which were previously shown to transmit between ferrets in a direct contact setting but displayed differences in transmission via air: A/Minnesota/45/2016 (MN/45) A(H1N2)v virus transmitted between 3/3 ferret pairs, A/Ohio/09/2015 (OH/09) A(H1N1)v virus transmitted between 1/3 ferret pairs, while A/Ohio/02/2007 (OH/02) A(H1N1)v virus did not transmit via air between any of the ferret pairs (Table 1)^25–27^. To evaluate airborne virus shedding three ferrets per group were inoculated with each of the 3 viruses. Nasal washes were collected from inoculated ferrets every other day for 9 days post-inoculation (p.i.) and were analyzed to determine the levels of infectious virus and viral RNA. Simultaneously, aerosols were sampled from ferret cages for 2 h/day on days 1, 2, 3, and 5 p.i. using a two-stage cyclone aerosol sampler (BC 251 model) that separates particles based on size into three fractions: >4 µm, 4–1 µm, and <1 µm^24^. Infectious virus was detected in nasal wash specimens for all viruses up to day 5 p.i., while viral RNA was detected through day 9 p.i. Viral RNA copy number averages were 1.9–3.7 orders of magnitude higher than average infectious virus titers detected between days 1 and 5 p.i. Comparable mean peak nasal wash titers were observed for MN/45 and OH/09 viruses (7.0 and 6.9 log_10_ PFU/mL, and 9.2 and 8.9 log_10_ RNA copies/mL, respectively), both of which were higher than observed peak titers for OH/02 virus (5.3 log_10_ PFU/ml and 8.1 log_10_ RNA copies/mL) (Fig. 1a, c, e). Despite high viral loads in the upper respiratory tract as measured in ferret nasal wash samples on day 1 p.i., negligible levels of viral RNA were detected in the air on day 1 p.i. (Fig. 1b, d. f). However, by day 2 p.i., high levels of viral RNA were detected in the air from all ferrets inoculated with MN/45 and OH/09 viruses. The viral RNA was collected primarily in the >4 µm fraction and was detectable for most ferrets through day 5 p.i. In contrast, the OH/02 virus RNA was not elevated in air samples until day 5 p.i. To determine whether the differences in replication kinetics and release of viral RNA into the air were statistically significant between the viruses tested, we compared area under the curve values for viral RNA copies detected in the nasal wash specimens and in the air collected through day 5 p.i. The results revealed that the viral RNA copy numbers in nasal washes as well as in the air samples collected from ferrets infected by OH/02 virus were significantly lower than for those infected by MN/45 virus, a highly transmissible virus (*p* = 0.02) (Fig. 1g, h). Collectively, these data show that collection of virus-laden aerosols shed by influenza virus-infected ferrets can provide meaningful contextual information regarding transmission capability, especially when collected early after inoculation.

**Table 1:** Transmissibility profiles of IAVs evaluated in this study.

Virus name	Name in the study	Subtype^a^	Virus origin^b^	Virus stock matrix^c^	DCT^d^	RDT^d^	Reference
A/Nebraska/14/2019	NE/14	A(H1N1)pdm09	Human	Cell	NT	3/3*	^5^
A/Idaho/07/2018	ID/07	A(H1N1)pdm09	Human	Cell	NT	3/3*	^5^
A/Michigan/45/2015	MI/45	A(H1N1)pdm09	Human	Cell	NT	3/3*	^5^
A/Ohio/09/2015	OH/09	A(H1N1)v	Swine^#^	Egg	3/3	1/3	^25^
A/Ohio/02/2007	OH/02	A(H1N1)v	Swine^#^	Cell	3/3	0/3	^26^
A/Michigan/288/2019	MI/288	A(H1N1)v	Swine^#^	Cell	3/3	3/3*	^5^
A/California/62/2018	CA/62	A(H1N2)v	Swine^#^	Cell	3/3	1/3*	^5^
A/Ohio/24/2017	OH/24	A(H1N2)v	Swine^#^	Cell	3/3	1/3*	^5^
A/Minnesota/45/2016	MN/45	A(H1N2)v	Swine^#^	Cell	3/3	3/3	^27^
A/Anhui-Lujiang/39/2018	Anhui-Lujiang/39	A(H9N2)	LPAI^#^	Egg	3/3	2/3*	^28^
A/turkey/California/18-031151-4/2018	tr/CA/18-031151-4	A(H7N3)	LPAI	Egg	1/3	NT	^30^
A/duck/Bangladesh/19D770/2017	dk/Bang/19D770	A(H5N6)	HPAI	Egg	1/3	NT	^29^
A/Yunnan/14563/2015	Yunnan/14563	A(H5N6)	HPAI^#^	Egg	0/3	NT	^29^
A/Sichuan/26221/2014	Sichuan/26221	A(H5N6)	HPAI^#^	Egg	1/3	NT	^29^

^a^Subtypes ending with “v” indicate “variant’ viruses (swine-origin virus isolated from a human).

^b^Human - human seasonal virus that emerged after 2009 pandemic; swine – swine origin virus isolated from a human; LPAI-low pathogenic avian influenza; HPAI-highly pathogenic avian influenza.

^c^Virus stocks were propagated in cells (Madin-Darby Canine Kidney) or 10–11 day old embryonated chicken eggs.

^d^Direct contact transmission (DCT) and Respiratory droplet contact transmission (RDT) outcome as determined by detection of infectious virus in nasal washes from contact ferrets.

^#^Zoonotic-origin viruses isolated from humans.

*Aerosol collection concurrent with RDT experimentation in the referenced study. *NT* not tested.

### Quantification of airborne viral RNA following inoculation of ferrets with IAVs that transmit via air

**Fig. 2:**
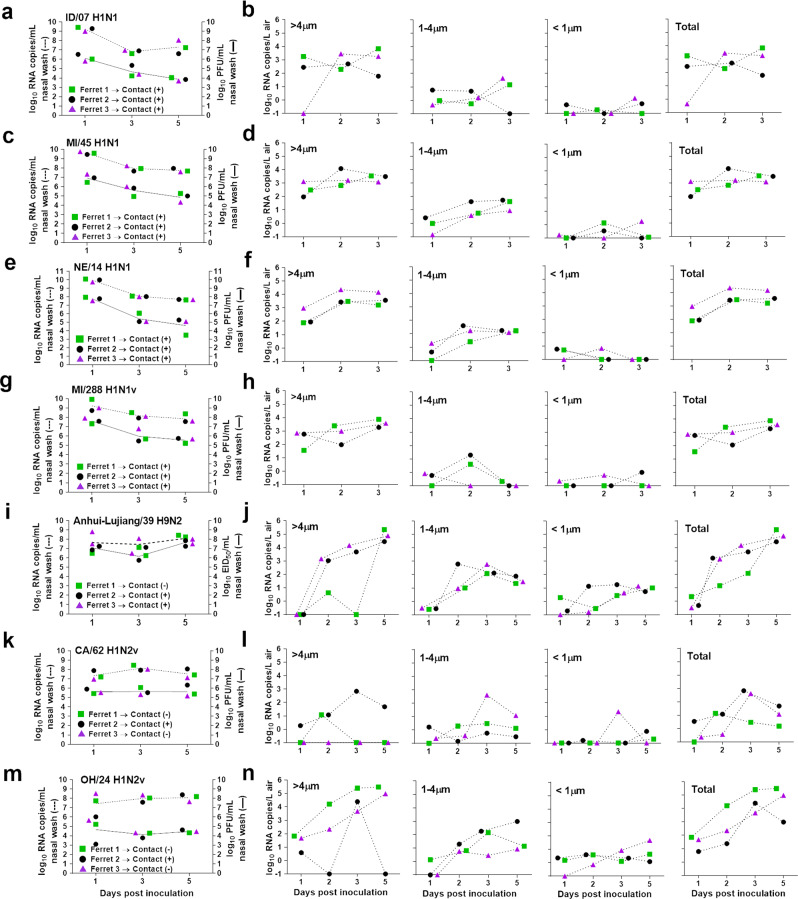
Influenza A viral RNA measured in the air during ferret transmission experiments. Three ferrets per group were inoculated with A/Idaho/07/2018 A(H1N1)pdm09 **a**, A/Michigan/45/2015 A(H1N1)pdm09 **c**, A/Nebraska/14/2019 A(H1N1)pdm09 **e**, A/Michigan/288/2019 A(H1N1)v **g**, A/Anhui-Lujiang/39/2018 A(H9N2) **i**, A/California/62/2018 A(H1N2)v **k**, A/Ohio/24/2017 A(H1N2)v **m**. The following day a naïve ferret was placed in an adjacent cage allowing for air exchange via perforated cage walls to assess airborne transmission. Infectious virus in nasal wash samples was quantified using plaque assay or by egg titration (solid lines; data source listed in Table 1; limit of detection 1 log_10_ PFU/ml or 1.5 log_10_ EID_50_/ml), and viral RNA copies were quantified using real time qRT-PCR (dashed lines; data generated in this study; limit of detection 1 log_10_ RNA copies/ml). Individual ferrets are shown as different symbols (ferret 1-green square, ferret 2-black circle, ferret 3-purple triangle). The plus sign indicates that infectious virus was detected in nasal wash samples collected from contact animals by day 5 post-contact; the negative sign indicates that transmission did not occur as confirmed by lack of the virus in nasal wash samples and by lack of seroconversion (Transmission data source listed in Table 1). Air samples were collected from each ferret cage for 2 h on indicated days using a two-stage cyclone sampler which fractioned particles by size: >4 µm, 1–4 µm, <1 µm (perforated cage side wall was blocked during air sampling). Viral RNA in each sample was quantified using real time qRT-PCR and the data for each virus; A/Idaho/07/2018 A(H1N1)pdm09 **b**, A/Michigan/45/2015 A(H1N1)pdm09 **d**, A/Nebraska/14/2019 A(H1N1)pdm09 **f**, A/Michigan/288/2019 A(H1N1)v **h**, A/Anhui-Lujiang/39/2018 A(H9N2) **j**, A/California/62/2018 A(H1N2)v **l**, A/Ohio/24/2017 A(H1N2)v **n**, and individual ferrets is shown (limit of detection 0.1 RNA copy/L of air).

Next, we sought to quantify the levels of viral RNA released into the air by inoculated ferrets that were housed in cages adjacent to contact ferrets during experiments utilizing the RDT model. Perforated sidewalls adjoining the cages were blocked during air collection to prevent collecting air from the contact ferrets. Viruses examined included three human seasonal influenza viruses [A/Idaho/07/2018 (ID/07) A(H1N1)pdm09, A/Michigan/45/2015 (MI/45) A(H1N1)pdm09, A/Nebraska/14/2019 (NE/14) A(H1N1)pdm09], three swine-origin influenza viruses [A/Michigan/288/2019 (MI/288) A(H1N1)v, A/California/62/2018 (CA/62) A(H1N2)v, A/Ohio/24/2017 (OH/24) A(H1N2)v], and one low pathogenic avian influenza (LPAI) virus [A/Anhui-Lujiang/39/2018 (Anhui-Lujiang/39) A(H9N2)]. Detailed information about clinical signs of infection as well as transmission and pathogenesis data can be found in references provided in Table 1. ID/07, MI/45, NE/14, and MI/288 viruses transmitted efficiently through the air between 3/3 ferret pairs by day 5 post-contact, Anhui-Lujiang/39 virus transmitted by day 3 p.c. between 2/3 ferret pairs, while CA/62 and OH/24 viruses transmitted by day 3 p.c. between 1/3 ferret pairs as evidenced by detection of infectious virus in nasal wash samples^5,28^. Viral RNA copy levels in nasal washes from animals inoculated with viruses that transmitted efficiently through the air (ID/7, MI/45, NE/14, and MI/288 peaked on day 1 p.i. and ranged between 9.3 and 10.0 log_10_ RNA copies/mL, which was 2–3 orders of magnitude higher than infectious virus (Fig. 2a, c, e, g). Viruses that transmitted inefficiently via air between ferrets (CA/62, OH/24, and Anhui-Lujiang/39) peaked variably between day 1 and 5 p.i., and the mean peak titers were 8.2–8.5 log_10_ RNA copies/mL (Fig. 2i, k, m).

For most ferrets inoculated with ID/7, MI/45, NE/14 or MI/288 virus, airborne viral RNA was detected at quantities ≥2 log_10_ RNA copies/L of air starting on day 1 p.i. and was maintained at 2-4 log_10_ RNA copies/L on days 2 and 3 p.i. (Fig. 2b, d, f, h). Animals inoculated with the Anhui-Lujiang/39 virus or CA/62 virus released negligible quantities of viral RNA into the air on day 1 p.i. (Fig. 2j, l). The two ferrets (Ferret 2 and 3) inoculated with Anhui-Lujiang/39 virus that released 3–4 log_10_ RNA copies/L on days 2–5 p.i. transmitted virus to their respective contact ferrets. Ferret 1, which released <1 log_10_ RNA copies/L on days 1–3 p.i., and 5.3 log_10_ RNA copies/L on day 5 p.i., did not transmit virus to the contact animal. Transmission was not observed from donor ferrets 1 and 3 that were inoculated with CA/62 virus and released ≤1 log_10_ RNA copies/L on days 1–5, while transmission did occur for ferret 2 that released 1–2 log_10_ RNA copies/L on days 2–5. The OH/24-inoculated ferrets released high levels of viral RNA (up to 5.4 log_10_ RNA copies/L); however, transmission occurred only between one pair (Ferret 2, Fig. 2n), highlighting that other factors, in addition to the amount of airborne virus, play an important role in the transmission of zoonotic influenza viruses. Collectively, these data show that the animals releasing high quantities of viral RNA in the air at early time points post-inoculation were most frequently associated with transmission events to contact ferrets, whereas animals that released low levels of viral RNA in the air or that shed peak levels at later time points post-inoculation, were less likely to transmit virus to contact ferrets in this setting.

### Quantification of airborne viral RNA following inoculation of ferrets with IAVs that poorly transmit

**Fig. 3:**
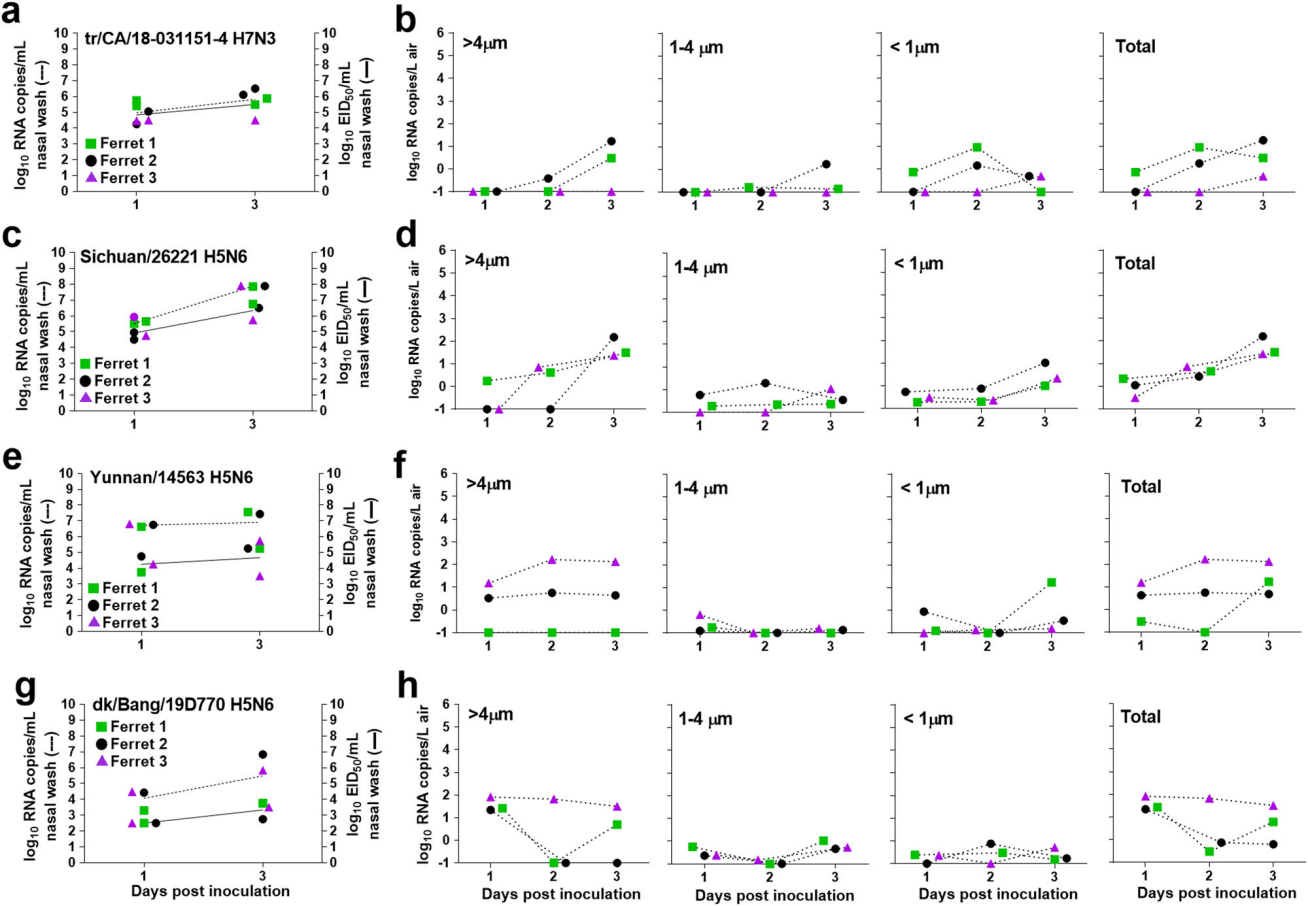
Quantification of airborne viral RNA released from ferrets inoculated with poorly transmissible influenza A viruses. Three ferrets per group were inoculated with A/turkey/California/18-031151-4/2018 A(H7N3) **a**, A/Sichuan/26221/2014 A(H5N6) **c**, A/Yunnan/14563/2015 A(H5N6) **e**, and A/duck/Bangladesh/19D770/2017 A(H5N6) **g**. Infectious virus in nasal wash samples was quantified by egg titration (solid lines; data source listed in Table 1; limit of detection 1 log_10 _EID_50_/ml), and viral RNA copies were quantified using real time qRT-PCR (dashed lines; data generated in this study; limit of detection 1 log_10_ RNA copies/ml). Each individual ferret is shown as a different symbol (ferret 1-green square, ferret 2-black circle, ferret 3-purple triangle). Air samples were collected from each ferret cage for 2 h on indicated days using a two-stage cyclone sampler which separated the aerosol particles based on size into three fractions: > 4 µm, 1–4 µm, <1 µm. Viral RNA in each sample was quantified using real time qRT-PCR and the data for each virus; A/turkey/California/18-031151-4/2018 A(H7N3) **b**, A/Sichuan/26221/2014 A(H5N6) **d**, A/Yunnan/14563/2015 A(H5N6) **f**, and A/duck/Bangladesh/19D770/2017 A(H5N6) **h**, and the individual ferret is shown (limit of detection 0.1 RNA copy/L of air).

To further expand our analysis, we next evaluated a panel of viruses that do not transmit or poorly transmit in the ferret model. As reported elsewhere (Table 1), the highly pathogenic avian influenza (HPAI) A/Yunnan/14563/2015 (Yunnan/14563) A(H5N6) virus did not transmit between co-housed ferrets, while the LPAI A/turkey/California/18-031151-4/2018 (tr/CA/18-031151-4) A(H7N3) and HPAI A/Sichuan/26221/2014 (Sichuan/26221) and A/duck/Bangladesh/19D770/2017 (dk/Bang/19D770) A(H5N6) viruses transmitted between 1/3 co-housed contact animals^29,30^. To investigate if reduced shedding of airborne viral RNA early after infection coincided with the lack of robust transmission among ferrets, we collected air from inoculated ferrets on days 1-3 p.i. (the period identified as most critical for transmission, Fig. 2). Out of the four viruses tested, ferrets inoculated with tr/CA/18-031151-4 virus displayed the lowest nasal wash RNA titers (5.9 log_10_ copies/mL) and released negligible levels of viral RNA into the air at ≤1.2 log_10_ copies/L on day 3 p.i. in the >4 µm fraction and <1 log_10_ copies/L in the other fractions (Fig. 3a, b). Ferrets inoculated with Sichuan/26221 virus also showed a delay in viral RNA release into the air with total titers up to 2.2 log_10_ RNA copies/L observed on day 3 p.i., which coincided with increased nasal wash titers observed at this later time point (mean peak of 7.9 log_10_ RNA copies/mL) (Fig. 3c, d). In the case of Yunnan/14563 virus-inoculated ferrets, 2/3 ferrets released 0.5–2.2 log_10_ RNA copies/L of air in the >4 µm fraction while the third ferret shed viral RNA in other fractions. Despite the differences in Yunnan/14563 RNA release into the air, comparable nasal wash titers were observed for the three ferrets with a mean peak at 7.4 log_10_ RNA copies/mL (Fig. 3e, f). Each of the three dk/Bang/19D770 virus-inoculated ferrets released detectable viral RNA into the air (≤2.0 log_10_ copies/L) by day 1 p.i.; however, only one of the ferrets maintained viral RNA shedding through days 2 and 3 p.i., while mean peak viral RNA levels in nasal wash were 6.4 log_10_ copies/ml from these ferrets (Fig. 3g, h). Unlike what was observed for the transmissible viruses, the avian strains tested here displayed less robust replication in the upper respiratory tract of ferrets, delayed replication kinetics, with low levels of viral RNA were released into the air within the first three days post-inoculation.

### Comparative analysis

**Fig. 4:**
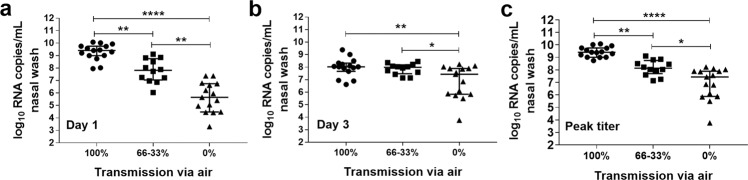
Comparison of viral RNA levels detected in nasal wash samples from ferrets inoculated with influenza A viruses displaying diverse transmission capabilities. Viral RNA copies in nasal wash samples collected from inoculated ferrets on day 1 p.i. **a**, day 3 p.i. **b**, or peak titer (either d1 or d3 p.i.) **c** are shown. Data were grouped by virus transmissibility profile in the ferret model (100%-transmission between all ferret pairs [A/Idaho/07/2018 A(H1N1)pdm09, A/Michigan/45/2015 A(H1N1)pdm09, A/Nebraska/14/2019 A(H1N1)pdm09, A/Michigan/288/2019 A(H1N1)v, A/Minnesota/45/2016 A(H1N2)v (3 ferrets per virus; *n* = 15)], 66–33% - transmission between 2/3 or 1/3 ferret pairs [A/Anhui-Lujiang/39/2018 A(H9N2), A/California/62/2018 A(H1N2)v, A/Ohio/24/2017 A(H1N2)v, A/Ohio/09/2015 A(H1N1)v (3 ferrets per virus; *n* = 12)], 0% - no transmission via air or inefficient direct contact transmission if airborne transmission was not tested [A/Ohio/02/2007 A(H1N1)v, A/turkey/California/18-031151-4/2018 A(H7N3), A/Sichuan/26221/2014 A(H5N6), A/Yunnan/14563/2015 A(H5N6), A/duck/Bangladesh/19D770/2017 A(H5N6) (3 ferrets per virus; *n* = 15)]. Kruskal–Wallis one-way analysis of variance (ANOVA) with Dunn’s post-test were used to determine significant differences, *p < 0.05, **p < 0.01, ***p < 0.001, ****p < 0.0001. Data points represent individual ferrets. Error bars represent median with interquartile range.

Previous studies have shown that the frequency of transmission through the air to a contact ferret decreases with time underscoring the importance of robust virus replication at early time points after inoculation^15,31,32^. To determine whether there is a significant difference in nasal wash titers collected at early times points p.i. (day 1, day 3 or peak titer) between transmissible and non-transmissible viruses we classified the viruses in three groups based on their transmissibility profile and performed statistical analyses. The results revealed that regardless of time point analyzed, viral RNA levels in nasal washes collected from ferrets inoculated with viruses that transmitted with 100% frequency though the air (transmission between 3/3 ferret pairs; ID/07, MI/45, NE/14, MI/288, MN/45) were significantly higher when compared to the titers detected from ferrets inoculated with viruses that transmitted less efficiently (Fig. 4). The levels of RNA in nasal wash samples collected on both day 1 and 3 p.i. from ferrets inoculated with viruses that transmitted with 66-33% frequency (transmission between 2/4 or 1/3 ferret pairs; Anhui-Lujiang/39, CA/62, OH/24, OH/09) were also significantly higher than titers detected from ferrets inoculated with viruses in the 0% transmission efficiency group (OH/02, tr/CA/18-031151-4, Sichuan/26221, Yunnan/14563, dk/Bang/19D770).

**Fig. 5:**
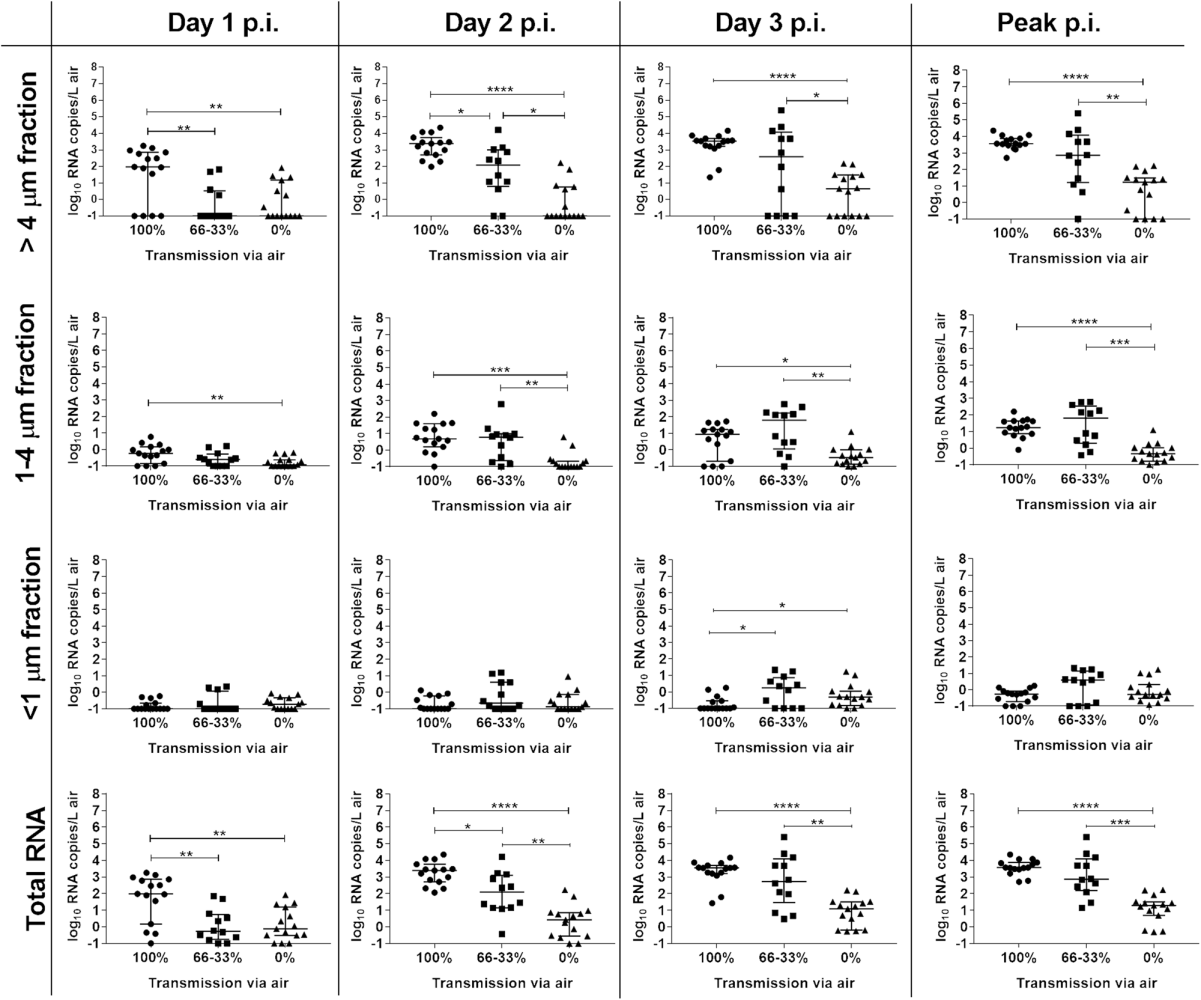
Detection of influenza A virus genomic RNA in size-fractioned air samples collected from ferrets. Viral RNA copies in particle size-fractionated air samples collected from inoculated ferrets on days 1-3 and peak viral RNA copies are shown (total RNA is inclusive of all size fractions). Viruses were grouped by transmissibility profile in the ferret model (100%-transmission between all ferret pairs [A/Idaho/07/2018 A(H1N1)pdm09, A/Michigan/45/2015 A(H1N1)pdm09, A/Nebraska/14/2019 A(H1N1)pdm09, A/Michigan/288/2019 A(H1N1)v, A/Minnesota/45/2016 A(H1N2)v (3 ferrets per virus; *n* = 15)], 66–33%—transmission between 1/3 or 2/3 ferret pairs [A/Anhui-Lujiang/39/2018 A(H9N2), A/California/62/2018 A(H1N2)v, A/Ohio/24/2017 A(H1N2)v, A/Ohio/09/2015 A(H1N1)v (3 ferrets per virus; *n* = 12)], 0%—no transmission via air or inefficient direct contact transmission if airborne transmission was not tested [A/Ohio/02/2007 A(H1N1)v, A/turkey/California/18-031151-4/2018 A(H7N3), A/Sichuan/26221/2014 A(H5N6), A/Yunnan/14563/2015 A(H5N6), A/duck/Bangladesh/19D770/2017 A(H5N6) (3 ferrets per virus; *n* = 15)]. Kruskal–Wallis one-way analysis of variance (ANOVA) with Dunn’s post-test was used to determine significant differences, *p < 0.05, **p < 0.01, ***p < 0.001, ****p < 0.0001. Data points represent individual ferrets. Error bars represent the median with interquartile range.

Next, we similarly analyzed the data obtained from air samples. Consistent with nasal wash RNA titer data, ferrets infected with transmissible viruses released significantly more viral RNA into the air than non-transmissible viruses at early timepoints p.i. (Fig. 5). The differences were more pronounced for viral particles collected in the >4 µm fraction, though some of these trends were also observed for particles 1–4 µm in size. In contrast, the viruses that displayed no or limited transmission were found at significantly higher levels in <1 µm fractions collected on day 3 p.i., indicating that this group of viruses may be aerosolized at later time points after inoculation from different parts of the ferret respiratory tract. Nonetheless, the overall levels of viral RNA in these fractions were much smaller as compared to other fractions. These data indicate that transmissible viruses have an upper respiratory tract replication advantage, thus achieving high replication peaks faster and releasing higher quantities of virus into the air, while viruses that are not capable of transmission via the air, in general, display delayed and lower nasal wash titers and peak titers in air samples.

## Discussion

The ferret is a valuable model of influenza virus transmission in humans and is routinely used for risk assessment of emerging strains. While it is well documented that human seasonal influenza viruses transmit more efficiently than most zoonotic viruses in this model, little is known about the kinetics of virus release into the air. To assess this parameter, we evaluated 14 divergent influenza A viruses in ferrets and measured the levels of virus shed into nasal wash samples and into the air collected from cages housing inoculated animals. As expected, human adapted A(H1N1) subtype viruses displayed robust replication in the upper respiratory tract with peak titers in nasal washes on day 1 p.i. Viral RNA released into the air from inoculated ferrets was detected as early as day 1 p.i., with transmission to respective contact animals in most cases by days 1–3 post contact. Out of the 11 zoonotic influenza viruses examined, only one swine-origin virus [A(H1N1)v MI/288] displayed high levels of replication in the upper respiratory tract concurrent with detection of viral RNA in the air by day 1 p.i. and transmission to all contacts^5^. These highly transmissible influenza viruses had two common traits: robust replication in the ferret upper respiratory tract as evidenced by the highest levels of viral titers detected at early times p.i., and early release of viral RNA into the air at levels sufficient for transmission to all contact animals. These traits varied for zoonotic viruses; for example, the highly transmissible MN/45 virus replicated to high levels in the ferret upper respiratory tract by day 1 p.i.; however, viral RNA was not detected in the air until day 2 p.i. Similarly, delayed release or low level of viral RNA in the air at early times p.i. was observed for most of the swine and avian-origin viruses that were evaluated in this study.

Delayed replication was most apparent for influenza viruses that poorly transmit between ferrets. This group of viruses, especially the avian-origin viruses, replicated to significantly lower levels at early times p.i. and were not able to reach comparable peak titers in the air as the transmissible influenza viruses, further supporting the utility of aerobiological techniques in the identification of zoonotic viruses with increased transmission potential. Differences in the abilities of transmissible and poorly transmissible influenza viruses to replicate in the upper respiratory tract of ferrets could be in part due to differences in receptor binding preferences. While human-adapted influenza viruses that transmit well preferentially bind α2,6 SA receptors, zoonotic influenza viruses, especially avian-origin, have a binding preference to α2,3 SA receptors, which are enriched in the lower respiratory tract^33^. Compelling evidence supports the importance of upper respiratory tract cells in promoting the selection of viruses with α2,6 SA binding preference^34^ as well as facilitating virus release into the air and onward transmission^20,21^. Future assessments of receptor binding specificity could potentially explain the lack of efficient transmission of viruses such as the OH/24 virus, which despite robust replication in the ferret upper respiratory tract and the presence of high RNA levels in the air, was not able to transmit via the air. Since the frequency of airborne transmission between ferrets has been reported to be highest at earlier time points p.i., before the onset of clinical signs of infection,^15,31,32^, delayed shedding of the virus into the air may further reduce the likelihood for transmission. In addition, localized, strain-specific differences in the activation of the immune response by IAVs exhibiting diverse transmissibility profiles in ferrets have been previously shown to correlate with peak virus shedding and the frequency of transmission^35^ warranting further evaluation of the interplay between immunological factors that are triggered upon infection and the kinetics of virus release into the air.

Influenza virus-laden particles of different sizes have been detected in human breath or cough^36–39^. The ferret model was subsequently employed to dissect the relative significance of droplets and fine droplet nuclei in mediating influenza transmissions. Zhou et al. (2018) showed that although 76.8% of aerosol particles released from an infected ferret were <1.54 µm, most of the viral RNA was detected in the >4 µm particle size and airborne transmission efficiency was abolished when ferret exposure was limited to ≤1.5 µm particles^15^. Recovery of viable virus from inoculated ferrets was also shown to be more efficient from fractions containing larger particles (>4.7 µm)^14^. Most of the viral RNA detected in sampled air during this study was found in the >4 µm particle fraction, which agrees with results from other influenza virus studies^15,16^. Our observation that particles of <1 µm contained negligible quantities of viral RNA especially for transmissible viruses further validates the role of >1 µm particles in influenza transmission in the ferret model through further investigation of the role smaller particle sizes contribute towards transmission events in this model is warranted especially when investigating diverse influenza virus subtypes.

A caveat of this study is that viral nucleic acid was measured in air samples, and infectious virus titration remains to be determined. Various collection techniques and devices can help preserve infectious virus, e.g. cascade impactors, multi-stage liquid impingers, and water-based condensation samplers, and offer important contextual information; however, experimental parameters and scalability considerations precluded their use in this study^11,13^. Previously, low levels of infectious virus were detected from the breath of anesthetized ferrets by using a cascade impactor^14^. Although the quantities of infectious virus were at least 1 order of magnitude lower than the collected RNA, differences in both infectious virus and viral RNA loads in the air were observed for a representative transmissible and a non-transmissible strain used in that study. The differences in infectious virus in nasal wash samples analyzed here were also lower than viral RNA, indicating that the overall levels of infectious virus released into the air are smaller than the observed viral RNA levels. A recent study capturing virus-laden particles directly on cell monolayers followed by quantification of plaque forming units revealed that ferrets inoculated with H1N1 virus emitted as much as 138 PFU/minute during peak of infection^17^. Considering that inhalation of less than 5 infectious particles can initiate a productive infection in a naïve host, the low levels of infectious virus in the air are sufficient for transmission to occur^40–42^. Ultimately, studies employing a range of collection devices can collectively provide critical insight into the role aerosols contribute to transmission. Additionally, virus stability in the air, a parameter that was previously shown to differ among virus strains^17,41,43^, as well as the composition of respiratory secretions during infection may also affect the overall levels of infectious virus in the air^44^. It should also be noted that persistence of virus in the environment, such as on bedding and ferret fur, could possibly contribute to the levels of viral RNA detected in the air and influence transmission frequency. Continued research is needed to elucidate the source of airborne particles generated during animal experiments and their relative role in airborne transmission.

In conclusion, quantification of airborne influenza virus RNA represents a valuable addition to established protocols used to evaluate transmission potential of newly emerging influenza viruses. The levels of influenza virus RNA collected in nasal wash and air samples from infected ferrets significantly differed between transmissible and non-transmissible viruses highlighting the utility of such assessments in quick identification of zoonotic influenza virus strains that may have enhanced transmission potential. Additional research is needed to correlate specific levels of virus (infectious and viral RNA copy numbers) in the air with airborne transmission in ferrets while taking into consideration virus- and host-specific parameters that facilitate efficient replication and emission of the virus into the air. A more comprehensive understanding of ferret-to-ferret transmission dynamics will aid in the development of predictive mathematical and statistical models to guide study designs with reduced number of animals used in accordance with the three R’s governing ethical animal research activities^45^.

## Methods

### Viruses

All influenza A viruses employed in this study are described in Table 1. Virus stocks were propagated in Madin-Darby Canine Kidney (MDCK) cells or 10–11 day old embryonated chicken eggs as specified in the references and Table 1. All experiments were conducted in BSL-3/ABSL-3 laboratories, with enhancements required by the US Department of Agriculture and the Federal Select Agent Program.

### Ferret inoculation and transmission experiments

All experiments were performed with the approval from the Centers for Disease Control and Prevention’s Institutional Animal Care and Use Committee (IACUC) and were conducted in an Association for Assessment and Accreditation of Laboratory Animal Care International-accredited animal facility. Ferret pathogenesis and transmission data were published previously as indicated by references in Table 1 unless otherwise specified. Male Fitch ferrets (6–11 months of age), serologically negative by hemagglutination inhibition assay to currently circulating influenza A (H1, H3), and B viruses, were employed in each study. The ferrets were anesthetized [intramuscular injection of a ketamine cocktail (20 mg/kg Ketamine, 0.05 mg/kg Atropine, 2 mg/kg Xylazine)] and then inoculated intranasally with 6 log_10_ PFU or EID_50_ of virus diluted in 1 ml of PBS and observed daily for clinical signs of infection. Nasal wash specimens were collected after air sampling; briefly, following anesthesia 1 ml of PBS was introduced into the ferret nasal passages to induce sneezing and the aspirate was collected in a sterile Petri dish^46^. The Direct Contact Transmission model (DCT) was established by co-housing a naïve ferret with an inoculated ferret (3 ferret pairs per virus tested) and the Respiratory Droplet Transmission model (RDT) was established by housing naïve and inoculated ferrets in adjacent cages separated by a perforated wall (3 ferret pairs per virus tested)^40^. The ferrets were housed in stainless steel cages within a Duo-Flo BioClean mobile isolator (Lab Products, Seaford, DE; uniform air flow circulated at 150–180 air changes per hour) throughout each experiment, including air sampling.

### Aerosol collection procedure

Aerosol samples were collected using a National Institute for Occupational Safety and Health (NIOSH) BC 251 two-stage cyclone aerosol sampler^24^. During experimentation each inoculated ferret was housed in a separate stainless-steel cage (56 cm L × 42 cm W × 42 cm H). For the respiratory droplet transmission experiments, the inoculated ferrets were housed in cages with the same dimensions but with a perforated side wall which was blocked during air sampling. The samplers were attached to the outside of each cage (cage door) with the sampler inlet protruding 3–4 cm into the cage. The sampler position in relation to the cage was the same in each sampling experiment. The air was collected at 3.5 L/min for 2 h, at the same time each day. The first sampler stage collected particles >4 µm into a disposable 15-mL collection tube (35-2096; Falcon), the second sampler stage collected particles with a diameter of 1–4 µm into a disposable 1.5-mL tube (02-681-339; Fisher Scientific), and the 37-mm polytetrafluoroethylene filter with 3 µm pores (FSLW03700, Millipore) collected particles with a diameter <1 µm. Following collection, samplers were disassembled in a Class II biological safety cabinet and viral material was immediately collected in PBS and inactivated in AVL buffer (Qiagen) according to the manufacturers protocol for subsequent RNA extraction and quantification. Samplers were decontaminated with 70% ethanol, submerged in water, and then rinsed thoroughly with distilled water and air dried after each sampling day. For experiments depicted in Fig. 1, ferrets were transferred to a clean cage before sampling on day 2; in all subsequent experiments, each ferret remained in the same cage throughout the air sampling time course.

### Virus quantification

To detect infectious virus, previously frozen ferret nasal wash specimens were titered by standard plaque assay in MDCK cells^47^. To detect and quantify viral RNA copy numbers, total RNA was extracted from samples using the RNeasy Mini Kit (Qiagen). Real-time RT-PCR was performed with a SuperScript III Platinum One-Step qRT-PCR System (Invitrogen) in duplicate reactions using an influenza A virus M1 gene primer and probe set (Influenza A Forward Primer: GAC CRA TCC TGT CAC CTC TGA C; Influenza A Reverse Primer: AGG GCA TTY TGG ACA AAK CGT CTA; Influenza A Probe FAM—TG CAG TCC TCG CTC ACT GGG CAC G—BHQ)^48^. Influenza virus M gene RNA copy numbers were extrapolated using a standard curve based on samples of known M gene copy number.

### Statistics and reproducibility

Data were collected from three ferrets per virus group in each experiment. For comparative analyses, the viruses were grouped by virus transmissibility profile in the ferret model (*n* = 12–15). All statistical analyses were performed using GraphPad Prism 6.0 software. The specific tests used are described in figure legends.

### Reporting summary

Further information on research design is available in the Nature Portfolio Reporting Summary linked to this article.

## Supplementary information

Description of Additional Supplementary Files

Supplementary Data 1

reporting summary

## Data Availability

The datasets generated during and analyzed during the current study are available in Supplementary Data 1.
