# Supplementary material for: Kinetics and magnitude of viral RNA shedding as indicators for Influenza A virus transmissibility in ferrets
Source: Commun Biol. 2023 Jan 23;6:90. doi: 10.1038/s42003-023-04459-0 (PMC9871019; doi:10.1038/s42003-023-04459-0)
Supplement: Supplementary file 1 — Description of Additional Supplementary Files [file 42003_2023_4459_MOESM1_ESM.pdf]

Description of Additional Supplementary Files

Supplementary Data 1 – Raw data for figures 1-5
